# Can Group Intelligence Help Entrepreneurs Find Better Opportunities?

**DOI:** 10.3389/fpsyg.2019.01141

**Published:** 2019-05-17

**Authors:** Yan Zichu

**Affiliations:** Department of Leadership and Organization Management, Tsinghua University, Beijing, China

**Keywords:** entrepreneurial opportunity, joint decision making, constructive controversy, industry environmental dynamism, entrepreneurship, group intelligence

## Abstract

Entrepreneurial activities are becoming more and more prevalence in our social life. One of the important questions in entrepreneurship is how to find good quality entrepreneurial opportunities. Previous researches suggested that characteristics of entrepreneurs such as their prior experiences, social capitals, and professional skills may influence the consequence of entrepreneurial opportunities finding. This research will introduce a more dynamic perspective to explain the influencing factor of the entrepreneurial opportunities finding. During the decision making process, some behaviors of team members such as joint decision making and constructive controversy may help decision makers understand key issues more comprehensive and decrease the risk come from uncertainties. Besides, this research also takes the different industries’ environmental dynamism into consideration. Thus, we can observe the internal and external effect at the same time.

## Introduction

With the economy developing these years, entrepreneurship activities have been paid more and more attentions to by researchers and managers. People want to reveal the myth of entrepreneurship and find the way to be successful. In most cases, entrepreneurs are the critical part of the entrepreneurship activities. Since 1980s, most entrepreneurship activities not only involve one entrepreneur but also teams doing the work. As Gartner et al. (1994) mentioned that “entrepreneur in entrepreneurship” is typically plural, not singular. Besides, according to the statistics of Panel Study of Entrepreneurship Dynamics (PSED), over half of the nascent entrepreneurs started their business with two or more partners (Reynolds et al., 2004). And PSED in China (2012) suggests that over sixty percent entrepreneurs realize the importance of establishing entrepreneurial teams when starting their business. Kamm, Shuman, Seeger and Nurick in 1990 firstly gave a strict definition for the entrepreneurial team. According to their words, entrepreneurial team should consist of at least two individuals and these individuals can get financial benefits from the new firm respectively. The entrepreneurial team phenomenon is significant because it is not only a prevalence phenomenon nowadays but also a critical factor to influence the consequence of entrepreneurship (Kamm et al., 1990). Compared with singular entrepreneur, entrepreneurial teams have the advantage of skills and background diversity. It is much easier for entrepreneurial teams to integrate the resources they have (Davidsson and Honig, 2003). Furthermore, entrepreneurial activities are always full of uncertainties and entrepreneurial team can cope better with these uncertainties (Niu et al., 2011). The better adaption ability brought by the entrepreneurial team can benefit entrepreneurial performance (Lechler, 2001). In practice, venture capitals also consider the entrepreneurial team as a critical factor to determine whether to invest a firm or not (Maschke and zu Knyphausen-Aufseβ, 2012).

Previous researches have suggested that successful new venture management cannot be reduced to a set of simple rules or techniques (Forbes et al., 2006). Entrepreneurial teams become successful because they not only have several people but also have a reasonable process to make each team member’s knowledge and resources function most effectively. Preceding studies about entrepreneurial teams most focus on the diversity in the team. These studies clarify that diversity especially in skills can make entrepreneurial team perform better. Some researchers find that team members’ entrepreneurial related experiences will influence entrepreneurial activities positively (Shrader and Siegel, 2007). Shu et al. (2018) points out that making full use of entrepreneurial team members can expand the social network resources of entrepreneurial teams, which is beneficial to entrepreneurial performance. Other studies major in dynamic team formation process also suggested that teams absorb new team members who own different skills, knowledge or background during the entrepreneurial activities (Ucbasaran et al., 2003). In contrast, some other studies mentioned that diversity in teams may impede the communications in teams because different people have different background knowledge would focus differently on the same thing, and that thus this may hinder team work efficiently.

Diversity can just provide some important resources for a new firm, but more important is how to use these resources. Rather than explaining whether diversity in entrepreneurial teams will influence the consequence of entrepreneurship as most of preceding researches have done, this study will try to reveal how entrepreneurial teams can take advantage of these diversities. In the setup stage of entrepreneurial activities, entrepreneurs should make two-thirds more decisions than managers in mature companies in average and decisions in entrepreneurial firms will more directly influence the future development of the firm. Thus, this research will focus on the perspective of constructive controversy and joint decision making in decision making process in entrepreneurial teams, and will explain how the process may affect entrepreneurial consequences. Meanwhile, in order to make the result more persuasive, we also take the opportunity differentiation in different industries when we evaluate entrepreneurship opportunities.

### Theoretical Background

Entrepreneurial opportunities are always core topic in the field of entrepreneurship researches. Plenty of scholars and practitioners regard entrepreneurial opportunities as one of the most important consequences in the entrepreneurial activities. Hansen et al. (2011) concluded the definitions of entrepreneurial opportunities in papers published in six most reputed journal in entrepreneurship field in the past nearly 20 years, and found that there are 49 conceptual definitions and 32 operational definitions about entrepreneurial opportunities. The most prevalence definition is given by Shane and Venkataraman (2000) as “situations in which new goods, services, raw materials, markets and organizing methods can be introduced through the formation of new means, ends or ends–means relationships… In addition, unlike optimizing or satisfying decisions, in which the ends that the decision maker is trying to achieve and the means that the decision maker will employ are given, entrepreneurial decisions are creative decisions. That is the entrepreneur constructs the means, the ends or both.”

Entrepreneurial opportunities make entrepreneurial teams clear about the direction of new firm development; thus, team members can devote their own advantages to solve certain problems. A great entrepreneurial opportunity not only means new, but also should be achievable and locate in an existing or potential big market. Most researchers agree that entrepreneurial opportunity is one of the key factors for a successful entrepreneurship. Previous studies about entrepreneurial opportunity most focus on the following question.

First is the classification of the entrepreneurial opportunity. Ardichvili et al. (2003) used two dimensions, value sought and value creation capability to classify entrepreneurial opportunity into four categories including “Dreams” “Problem Solving” “Technology Transfer” and “Business Formation.” This classification suggests that different kinds of entrepreneurial opportunities should have different risks and different potential benefits.

Second are the resources of entrepreneurial opportunities. There is a debate in the field of entrepreneurship that whether the entrepreneurial opportunities are recovery by entrepreneurs in the objective environment or created by the subjective recognition of entrepreneurs. Renko et al. (2012) integrated these two perspectives and proposed that entrepreneurial opportunity is objectively existing but should be explored by subjective recognition and behaviors of entrepreneurs.

Another critical issue is entrepreneurial opportunity recognition. Entrepreneurial opportunity recognition consists of two parts: the first part is the quantity of entrepreneurial opportunity entrepreneurs find and the second part is the quality of entrepreneurial opportunity. Most previous studies about entrepreneurial opportunity recognition focus on the quantity of opportunities. They operationalize entrepreneurial opportunity as the amount of opportunities entrepreneurs find in recent two or five years (e.g., Hills et al., 1997). But it is obviously not reasonable to judge entrepreneurial opportunity recognition solely from the perspective of quantity. Hansen et al. (2011) also criticized these kinds of operationalization. Timmons and Spinelli (1999) listed eight standards to evaluate quality of opportunities. Samuelsson (2004) in his doctoral dissertation gave out four criterions for innovation of opportunities. But entrepreneurial opportunity does not equal to innovation, and new ideas or methods do not always mean good. When we talk about entrepreneurial opportunities, we should also pay attention to the feasibility or operationalization of the opportunities and potential benefits the opportunities can bring. Rather than the opportunities self, the social environment and industry requirement also influence the evaluation of entrepreneurial opportunity.

As for the antecedents of entrepreneurial opportunity, most of preceding studies explain from the perspective of entrepreneurs’ characteristics or teams’ characteristics, such as entrepreneurs’ cognitive abilities (Corbett, 2007), human capital (Ucbasaran et al., 2009), social capital (Zhang et al., 2008) and confidence of entrepreneurs (Krueger and Dickson, 1994). But these factors are static and cannot reflect the dynamic influence on the quality entrepreneurial opportunity. Few studies pay attention to team process factors. Kollmann et al. (2018) investigate the relationship between task conflict and entrepreneurial capability in teams planning a business. In this study, scholars find task conflict is beneficial to business planning performance. However, they did not give a sufficient explanation to the team interaction process. George et al. (2016) did a systematic review of entrepreneurial opportunity recognition during past three decades. They suggest knowledge integration in teams is one of the important mechanisms to opportunity recognition, high quality knowledge integration is crucial to opportunity improving. Thus, following researches should more focus on the team process that can integrate knowledge from different team members. In order to cover shortages of opportunity quality evaluation and lack of dynamic process in previous study, this study will evaluate the quality of entrepreneurial opportunity according to the comprehensive suggestions of western scholars and some Chinese scholars and focus on the decision-making process which is critical for utilizing static resources. At the same time, we will also take external environment into consideration.

### Hypotheses Development

Entrepreneurial teams usually consist of individuals owning different backgrounds, experiences and skills. Some team members cannot clearly know the roles of themselves in the team or cannot understand well about the missions of the new firm. Thus, collaboration between the team members may encounter many problems (Foss et al., 2008). Entrepreneurships are activities full of uncertainty, and the quality of coordination among team members make this uncertainty become more serious to some extent. Blatt (2009) find that team members who are different in age, education and experience will be more likely to behave differently. More importantly, the different behaviors and understandings among team members may do harm to the normal function of the new firm if team members cannot communication well with others (Foss et al., 2008). Thus, cooperation among team members especially when in decision making becomes really important for an entrepreneurial team.

Joint decision-making means that team members participate into the process of decision making and that the final decision is not made by any one solely. Following the definition given by Subramani and Venkatraman (2003), we define the joint decision making as the degree to which team members jointly make decisions about key issues in the new firm. Joint decision making is not only a mechanism of cooperation but also a mechanism of mutual monitoring. Team members can take their own advantages to help the new firm prevent from risk actions or to reduce the cost of team learning. They can solve the problems in the entrepreneurial process together and avoid the loss brought by bias recognition of any certain individuals. As for mutual monitoring, joint decision making can prevent team members making decisions which are beneficial to individuals rather than the firm. More importantly, the process of joint decision making let core team members know well about the development of the firm, and it will make communication among team members more efficient and make team members more confident about the project they undertake. Novara et al. (2018) also suggests that the specific mode of problem solving should be made by the person face the problem directly, who may also find the opportunities behind the problems. In entrepreneurial teams, different team members usually responsible for different areas but these areas are highly interdependent, so joint decision-making process can help the team make more informed decisions.

Furthermore, joint decision making can also make team members feel that the process of decision making is fair (Kim and Mauborgne, 1993). The fairness feeling can make team members have higher potential to believe that the final decision is reasonable (Sapienza and Korsgaard, 1996) and have greater motivation to cooperate with other team members. Besides, fairness decision making process ensure that each team member can get the profit they should own. Team members getting the profit they deserved is a critical guarantee for their further devotions. Wang (1992) also mentioned that better joint decision making is beneficial to promote team performance. Thus, we propose that:

H1: Joint decision making is positively related to entrepreneurial opportunity evaluation, and the higher degree of joint decision making in team usually leads to better quality of entrepreneurial opportunity finding.

Another important factor that may influence the final decision-making is controversy in the decision-making process. Controversy is a special kind of conflicts. Team members discuss the different views, ideas and perspectives on the same question in public, and try to get consensus recognition and decision making. Tjosvold (1982) found that some kinds of controversy are beneficial to decision making, and he named those kinds of controversy as constructive controversy. Tjosvold (1982, 1984) also gave a definition of constructive controversy that constructive controversy refers to the open discussion on the different views for firm’s common interests. This discussion should be advantageous to team members’ explore thinking, finding new information and integrating different opinions among team members. Moreover, Gullo et al. (2015) emphasizes the important effect of positive working relationship in the team process. Constructive controversy on the one hand simulate cognitive conflict among team members, which can help team members consider issues comprehensively. On the other hand, constructive controversy aims at the problems rather than specific persons, thus constructive controversy can avoid emotional conflict that may harm to the positive working relationship.

Entrepreneurial activities are activities full of uncertain, and it is normal that there are different opinions existing in the team when team members understand this kind of uncertainty from different perspectives. Ou et al. (2018) discuss the relationship between constructive controversy and creative process engagement. They find constructive controversy can cultivate the positive conflict value and promote the creative engagement in the team. Entrepreneurial teams usually explore new fields or new mode of productions, thus team members engage in creative process is crucial to the success of the startups. Chen and Ning (2010) pointed out that constructive controversy can help team overcome the communication gap which caused by diversity of team members. Besides, Levinthal and March (1981) also suggested that none of individuals has unlimited cognitive and analysis ability, thus it is impossible to avoid systematic bias when team members make decisions depending on sole individual’s prior experiences. Constructive controversy can avoid individual’s cognitive bias in team’s critical decision making. Public discussion of different opinions can also prevent the team falling into aimless groupthink (Tjosvold and Johnson, 1983) and ensure the efficiency and quality of decision making. We therefore propose:

H2: Constructive controversy in the entrepreneurial team can help the team make better decisions, thus entrepreneurs can find entrepreneurial opportunities of higher quality.

Industry environmental dynamism is one of the most important external factors may influence the entrepreneurial opportunity evaluation. Dess and Beard (1984) defined environmental dynamism as the rate of unpredicted change within the industry that the startup operates. Existing research has proved that environmental dynamism can moderate the relationship between leadership style and entrepreneurial performance (Waldman et al., 2001). Yan and Liu (2018) use simulation experiment to explore the how environmental dynamics influence entrepreneurs’ opportunities recognition. The results suggest that in the case of more dynamic environment, information processing plays a stronger role in identifying better opportunities for entrepreneurs. Ensley et al. (2006) also pointed out that transformational leadership was most effective in dynamic environment.

**FIGURE 1:**
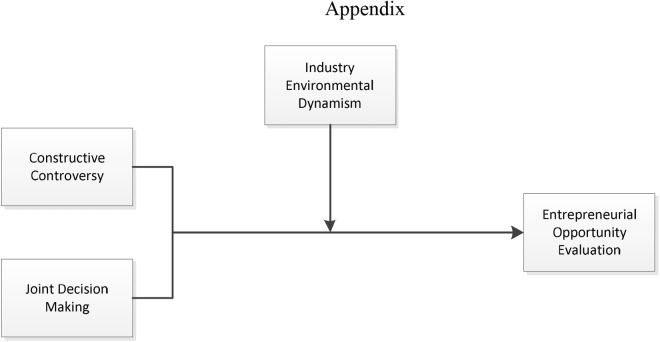
The integrated framework of the model.

Joint decision making and constructive controversy make entrepreneurial team have the ability to face uncertainty better. Compared with industry in stable environment, the new industry or industry in dynamic environment will let entrepreneurial firms face higher level and higher frequency of uncertainties. Thus, for entrepreneurial firms in high dynamic environment industry, the ability to response to uncertainties becomes more important. Therefore, we propose following two hypotheses (integrative model, see Figure 1):

H3a: Industry environmental dynamism can moderate the relationship between joint decision making and entrepreneurial opportunity evaluation. Higher level of industry environmental dynamism will magnify the effect of joint decision making on entrepreneurial opportunity evaluation.

H3b: Industry environmental dynamism can moderate the relationship between constructive controversy and entrepreneurial opportunity evaluation. Higher level of industry environmental dynamism will magnify the effect of constructive controversy on entrepreneurial opportunity evaluation.

## Materials and Methods

### Sample

We collected data from 259 individuals come from 82 entrepreneurial teams which participated in an entrepreneurial competition in Tsinghua University. We handed out our questionnaires before they started displaying their programs and took back the questionnaires when they completed the program displaying. Participation was voluntary, respondents were assured of the confidentiality of their responses, and the informed consent of the participants was implied through survey completion. All of participants have been informed that this survey is independent from the competition. The results of the survey would only keep for academic research rather than influencing the competition results. In order to avoid common method bias, we also get evaluations of programs from judges of the competition and evaluations of industry environmental dynamism from three doctoral students who are familiar with this field.

Of the 380 surveys were distributed, 259 complete surveys were returned, giving a response rate of 68.1%. We delete the teams which are less than three participants. Thus, the final valid data come from 207 individuals of 53 entrepreneurial teams. Of the participants, 22.2% were female, the average age of was 28.5 years (*SD* = 5.8), and the average team tenure was 9.7 months (*SD* = 3.2).

As for judges, there are five groups of judges in the competition. Each group consists of three judges. All judges are senior VC investors or professors major in entrepreneurial field. All of them have the experience of been judges in different kind of entrepreneurial competition for several times. Thus, their evaluation should be reasonable and professional.

One of the three doctoral students who evaluated Industry environmental dynamism is the author self and another two are the students major in entrepreneurial field and are familiar with entrepreneurial practice. Before they evaluated, they were asked to do preparation works for industry background again and be explained clearly about the mean of industry environmental dynamism.

### Measures

Part of survey items this study selected which were originally in English have been translated by some Chinese scholars before, thus we use the translated items directly. Others English items were translated into Chinese by two students following the commonly used back-translation procedure (Brislin, 1986).

#### Team Constructive Controversy

To measure constructive controversy, we adapted the six items scale developed by Chen et al. (2006) and translated into Chinese by Chen and Ning (2010). This is also one of the most common used scales to measure constructive controversy. Sample item is like “Team members can display their own opinions to others directly.” Participants rated each item on a scale from 1, “strongly disagree,” to 7, “strongly agree” (α = 0.86).

Because these items we directly measure on individuals we should aggregate to team level. We conducted ANOVA and calculated the value of ICC1. The ICC1 estimates for team constructive controversy was 0.29 (*p* < 0.05). Thus, we use average scores among team members to represent the score of team constructive controversy for an entrepreneurial team.

#### Joint Decision Making

To measure joint decision making, we used the three items scale developed by Subramani and Venkatraman (2003). Sample item is like “All team members can influence firm’s decision making.” Participants rated each item on a scale from 1, “strongly disagree,” to 7, “strongly agree” (α = 0.73).

Be same as team constructive controversy, we also need to aggregate this item into team level. ICC1 estimates for joint was 0.23 (*p* < 0.05). Thus, it is reasonable to use average scores of team members’ joint decision-making scores to represent the joint decision-making score of the team.

#### Entrepreneurial Opportunity Evaluation

The entrepreneurial opportunity evaluation is scored by judges of the competition. According to Timmons’ (1999) suggestion 11 standards of entrepreneurial opportunity evaluation and Wu and Wu (2011) suggestions for entrepreneurial opportunity evaluation standards in China, we choose seven standards to evaluate entrepreneurial opportunity in this study. These standards respectively are technology innovation, business model innovation, product or service innovation, product or service feasibility, potential market capacity, potential development of the program, team member construct. Each program has been evaluated by three judges. In order to avoid bias in the evaluation, we used average scores of three judges to represent final scores for entrepreneurial opportunity evaluation of the team.

#### Industry Environmental Dynamism

Industry environmental dynamism is evaluated by three doctoral students. According to the common industry classification of entrepreneurial competition nowadays, we classified programs into eight industries based on their application forms. These industries include: education, TMT, medical care, modern service industry, precision manufacturing, materials, environment and energy source and Industrial and architectural design. Following, Dess and Beard (1984) suggestion and Yang (2014) implication, we evaluate industry environmental dynamism majorly two perspectives: the amount of unpredicted issues happened in the industry recent years and the technical/business model changing recent years. Doctoral students rated each industry on a scale from 1, “most stable,” to 9, “most dynamic” (α = 0.76).

#### Control Variables

We put several control variables in our studies. Following previous researches, we controlled for team members’ average age, firm tenure (months), team size, average education level (1: below bachelor, 2: bachelor, 3: master,4: PhD), whether have any full time program participants (1: yes, 2: no), does any team member has entrepreneurial experience before (1: yes, 0: no). Besides, according to plenty of research about entrepreneurial activities suggestion, we also controlled the patents own condition of the teams (1: yes, 0: no).

### Analytic Strategies

Because of the multilevel nature of the data, we conducted hierarchical linear modeling (HLM) using HLM 6.08 to test our hypotheses (Raudenbush et al., 2004). Since the programs were from different industry, we included that an ordinary regression based may possible confounding effects of industry level factors on the relationship. Thus, we use two level models in our analysis with teams at level 1 and industries at level 2.

## Results

Table 1 shows the descriptive statistics among the study variables at the team level and industry level. In this table, we do not find surprising relationships among variables. Before we conducted HLM, we also tested the multi-collinearity of the data, and find no problems.

**Table 1:** Means, standard deviations, and correlations among variables.^a^

Variable	Means	*SD*	1	2	3	4	5	6	7	8	9	10	11
**Level 1**													
1. Age	28.75	5.60	1.00										
2. Education	2.88	0.59	0.37**	1.00									
3. Experience	1.14	0.33	-0.19	-0.19	1.00								
4. Fulltime	1.28	0.45	-0.41**	-0.14	-0.03	1.00							
5. Patent	0.70	0.49	0.07	0.22	0.03	-0.08	1.00						
6. Controversy	5.30	1.23	0.10	0.11	0.32*	-0.09	0.38**	1.00					
7. Joint decision	5.28	0.92	0.10	0.11	-0.03	0.18	0.31*	0.40**	1.00				
8. Team Size	5.32	2.23	-0.11	0.11	0.07	0.04	0.14	-0.11	-0.19	1.00			
9. Duration	9.60	5.17	0.19	-0.11	0.11	-0.08	0.33*	0.26	0.22	0.26	1.00		
10. Evaluation	75.07	8.65	0.18	0.24	0.10	0.01	0.52**	0.66**	0.62**	0.00	0.26	1.00	
**Level 2**													
11. Environmental Dynamism	5.62	1.18	0.25	0.32*	-0.02	-0.09	0.28*	0.45**	0.32*	0.01	0.14	0.41**	1.00


*^a^n = 53. ^∗^p ≤ 0.05; ^∗∗^p ≤ 0.01.*

### Hypothesis Tests

Hypothesis 1 suggested that teams’ joint decision making should positively relate to entrepreneurial opportunities evaluation. We put all control variables and level 1 predictors in the model 1. All predictors in the level 1 have been group mean centered. The results of model 1 in Table 2 shows that the team joint decision making was significantly related to entrepreneurial opportunities evaluation (*r* = 6.59, *p* < 0.01). Thus, Hypothesis 1 has been strongly supported.

**Table 2:** Hierarchical linear modeling results for entrepreneurial opportunity evaluation.

Variables	Model 1	Model 2	Model 3
Intercept	76.27**	76.27**	76.27**
Age	0.14	0.11	0.15
Education	0.10	0.06	0.16
Experience	3.03	3.21	2.44
Fulltime	0.80	0.59	0.49
Patent	3.73	3.70	3.60
Controversy	6.24*	4.93	6.76*
Joint decision	6.59**	6.83**	6.78**
Team Size	0.39	0.36	0.44
Duration	-0.01	0.00	-0.03
Interaction effect			
Controversy × Environmental Dynamism		-1.90	
Joint decision × Environmental Dynamism			1.76
Deviance	320.04	317.68	317.74


*n = 53. ^∗^p ≤ 0.05; ^∗∗^p ≤ 0.01. Two-tailed tests.*

Another main effect was also tested in model 1. Hypothesis 2 proposed that constructive controversy in the team can promote teams’ entrepreneurial opportunities evaluation. As Table 2 shown, the relationship between constructive controversy and entrepreneurial opportunities evaluation is significant (*r* = 6.24, *p* < 0.05). Therefore, the test supported Hypothesis 2.

To examine moderation effect, we conducted HLM in model 2 and model 3. In both models, we used group mean centered environmental dynamism scores (level 2 predictor). Hypothesis 3a predicted that in more dynamic industry environment, joint decision making will relate to entrepreneurial opportunity evaluation stronger. Model 3 suggests the coefficient of the cross term was not significant (*r* = 1.76, *p* > 0.05). Hypothesis 3b predicted the moderation effect on another main effect. But as shown in Table 2 (model 2), the coefficient is also not significant (*r* = -1.90, *p* > 0.05). Therefore, both Hypothesis 3a and Hypothesis 3b have not been supported.

## Discussion

In our study we find that joint decision making and constructive controversy are positive related to entrepreneurial opportunity evaluation. Two main effects both have been supported. But we can find that joint decision making has more significant effect on entrepreneurial opportunity evaluation than constructive controversy. This may because joint decision-making effect on final decision of firms more directly. Constructive controversy only refers to team members can display their own different opinions but it does not restrict when to display these opinions. The suggestions merge rightly during the decision-making process are more likely to influence decision makers’ ideas. Joint decision making may not only involve making suggestion, but also make suggestions at the right time. Thus, joint decision making has stronger effect on entrepreneurial opportunity evaluation seems reasonable.

None of our moderation prediction has been supported by this study. We consider there are following possible reasons can cause these failures. First is our sample size is not big enough and the amounts of teams in different industry are not balance. In this dataset, some industries include nearly 15 teams and some only include four teams. Because of the small sample size, each special case can have big influence on the final statistical result. Thus, the result may be not robust enough. The second possible reason is the real influential factor for entrepreneurial opportunity evaluation is not the objective industry environment dynamism, but the industry environment dynamism perceived by judges. Entrepreneurial opportunity evaluation is provided by judges based on their subjective perception. Although judges are professional investors and professors in this field, it is normal they are more familiar with some certain industries. This may lead to bias evaluations when judges observing programs. For the same reason, three doctoral students who scored the industry environment dynamism may also suffer this kind of bias. Therefore, we failed to prove the two moderation effect.

### Theoretical Implications

This research introduced more dynamic influencing mechanism on entrepreneurial opportunity evaluation than previous study. Most preceding studies solely focus on entrepreneurs’ or entrepreneurial teams’ statistical characteristics cannot explain well about why similar entrepreneurs will make different decisions. Combined with previous studies, this research suggested a resource integration mechanism which has highly influence on entrepreneurial consequence. Besides, most of previous researches on entrepreneurial team focus on the function of team leaders, few cares about other team members. This research emphasized the importance of team members.

Although our test failed support the moderation effect. The selection of industry environment dynamism also should be a good implication for academic researches. There are a lot of researchers in the entrepreneurial field mentioned that different industries may have different effect, but few of them pointed out why the effect should be different. This research suggested the reason why different industries are different is because their environment dynamisms are not the same. Only when we pointed out what cause the differences, can we know why it should be different.

### Practical Implications

This research suggested that make good use of team members’ intelligence can help entrepreneurial teams find better opportunity. For entrepreneurial teams, they usually have few resources and should face high level of uncertainties. Compared with mature firms the entrepreneurial firms are much more fragile, any tiny decision error may lead to the firm bankrupt. In order to consider more comprehensively before decision making, team leader should listen to the opinions of team members carefully and take these suggestions into consideration when making decisions.

Besides, let team members join in the decision-making process directly is also a good way for entrepreneurial teams. Joint decision making is one of the most efficiency methods to absorb team members’ intelligence. It can also make team members understand firms’ decisions more comprehensively so they can execute better. More important, invite team members to participate into decision making progress can also make them feel fairness atmosphere in the companies and they would have higher motivation to devote.

### Limitations and Future Researches

Although we make a lot effort to conduct this study, there are also a lot of limitations existing in the research. First, as we mentioned in the preceding part, the sample size may not big enough, this may influence our data analysis results. Second, the data is collected during the competition, team members may likely evaluate their teams better than they really like. Third, it is obviously that no matter joint decision making or constructive controversy will have certain cost, but we did not consider the dark side of joint decision making and constructive controversy. Fourth, although we want to reveal a dynamic mechanism, the observations of this study came from a point of time. It may make us ignore some important factors during the process.

In the future researches, we should try to find more complete mechanisms which influence entrepreneurial opportunity evaluation. For example, after decision maker receiving suggestions from team members, decision maker should have a process to deal with the suggestions. How decision maker evaluates the suggestions or whether there exists a process to polish these suggestions through repeated interaction with team members will all influence the final consequence. Furthermore, we suppose constructive controversy and joint decision making can promote entrepreneurial opportunity quality because they can reduce the uncertainties teams facing. But we did not test the whole path. Future researches can try to confirm whether the effect work really through the uncertainties reducing.

Besides, we also should do further studies about the content of industries’ differences. Many scholars and practitioners have realized that it may not reasonable to compare two entrepreneurial opportunities in different fields. But the problem is when can we compare two entrepreneurial opportunities? Do the entrepreneurial opportunities in the same industry is the only condition to conduct the comparison?

Or can we compare opportunities in different industries if they meet some requirements? Future in-depth researches may help us solve these problems.

## Conclusion

Entrepreneurial opportunity quality is a critical factor that influences the entrepreneurial consequences and all the people participate into entrepreneurial activities should notice how can find a better opportunity. This research suggests that joint decision making and constructive controversy among team members can help entrepreneurial teams find better entrepreneurial opportunities. Furthermore, this study also explains why joint decision making has stronger effect than constructive controversy.

## Ethics Statement

The data I used in this study all collect by my own and there is no relevant third party (A specific ethics approval was not required as per applicable institutional and national guidelines). During the data collection process, all the respondents are anonymous and they were informed all the data we collect is just for academic research purpose and be kept confidential (the respondents are clear that if they are willing to answer the questionnaire, it indicates that they agree with the purpose of the questionnaire). Furthermore, these data not involve biological information or other sensitive information, we confirm the data collection process and data analysis process would not cause any potential detriments to respondents or other parties.

## Author Contributions

YZ independently completed the survey design, data analysis and manuscript writing.

## Conflict of Interest Statement

The author declares that the research was conducted in the absence of any commercial or financial relationships that could be construed as a potential conflict of interest.
